# Algebraic method for multisensor data fusion

**DOI:** 10.1371/journal.pone.0307587

**Published:** 2024-09-27

**Authors:** Xiangbing Chen, Chen Chen, Xiaowen Lu

**Affiliations:** 1 School of Mathematics and Statistics, Kashi University, Kashi, Xinjiang, China; 2 Division of Mathematics, Sichuan University Jinjiang College, Meishan, Sichuan, China; 3 School of Science, Civil Aviation Flight University of China, Guanghan, Sichuan, China; The Hong Kong Polytechnic University, HONG KONG

## Abstract

In this contribution, we use Gaussian posterior probability densities to characterize local estimates from distributed sensors, and assume that they all belong to the Riemannian manifold of Gaussian distributions. Our starting point is to introduce a proper Lie algebraic structure for the Gaussian submanifold with a fixed mean vector, and then the average dissimilarity between the fused density and local posterior densities can be measured by the norm of a Lie algebraic vector. Under Gaussian assumptions, a geodesic projection based algebraic fusion method is proposed to achieve the fused density by taking the norm as the loss. It provides a robust fixed point iterative algorithm for the mean fusion with theoretical convergence, and gives an analytical form for the fused covariance matrix. The effectiveness of the proposed fusion method is illustrated by numerical examples.

## 1 Introduction

In recent decades, multisensor data fusion has been studied widely in many fields, such as target tracking, image processing, remote sensing and robotics [1–3]. The key to data fusion is to best utilize useful information contained in multiple sensors for the purpose of estimating an unknown quantity. Extensive research generally centers on two basic architectures, i.e., distributed fusion and centralized fusion. The former has received more attention due to less communication burden, higher reliability and flexibility, but is relatively more challenging for coping with unavailable correlations [4, 5].

In many cases, however, it is difficult to exactly know the correlations. The data fusion under uncertain correlations was first investigated with the covariance intersection (CI) method [6, 7], which utilizes the posterior mean and covariance. As a primitive CI version, the determinant-minimization CI (DCI) method [8] determines the normalized weights through minimizing the determinant or trace of estimation error covariance matrix. Considering the difference among the components of the local state estimate from each sensor, the CI fusion weighted by diagonal matrix (WDCI) [9] has higher fusion accuracy than the classical CI method. Recently, a linear estimation fusion method, termed DDF in [10], has been proposed for uncertain diagonal-constrained cross-covariance. The fused estimator is a linear unbiased combination of the posterior means whose weight coefficients are obtained by a semidefinite programming. Intuitively, complete probability density functions (PDFs) of local estimates are preferred to the first two moments for data fusion. The fast CI (FCI) method [11] minimizes Chernoff information to locate the “halfway” point between two local posterior PDFs, and has been generalized to deal with the case of more sensors. The Kullback–Leibler averaging (KLA) method [12] defines an average PDF as the one that minimizes the sum of KL divergences from local PDFs. In the context of fusing PDFs, more fusion methods are proposed in [13–18]. As it turns out, the PDF is more suitable for describing each local information.

It is well known that a typical problem in distributed data fusion is how to combine the point estimates of the quantity of interest with the corresponding mean-square error (MSE) matrices. When the local estimation information (an estimate and its MSE matrix) is represented as the PDF, the data fusion is performed on the space of PDFs with non-Euclidean geometrical structure [19, 20]. Information geometry regards the parametric space composed of PDFs as a Riemannian manifold, and studies information science using some basic geometrical quantities such as distance and curvature [21]. Apparently, the geometrical illustration develops an intrinsic understanding of statistical models, and also provides an intuitive perspective for estimation fusion. Under the information-geometric framework, the geodesic projection (GP) method [22] takes the fused PDF in the Gaussian manifold as an informative barycenter of all local posterior PDFs by minimizing the sum of their geodesic projection distances onto the Gaussian submanifold $N_{\mu }^{m}$ with a fixed mean ***μ***. In practice, the GP is an effective approach as its invariance when any affine transformation is done on the local estimates, but it has two major disadvantages. One is that the fused covariance estimate is not specifically designed, just adopting the same form as the CI, and the other is that the convergence of the GP cannot be guaranteed theoretically.

In the paper, we propose a novel algebraic fusion method, based on the Lie algebraic theory. The main contributions are listed as follows:

(i) By introducing a proper Lie algebraic structure for $N_{\mu }^{m}$, the dissimilarity between two Gaussian posterior densities can be evaluated with a Lie algebraic vector. Taking the norm of the average of Lie algebraic vectors as the loss, we construct an algebraic criterion for fusion.
(ii) The proposed fusion algorithm obtains the fused mean estimate by iteratively solving a fixed point equation, and then yields an explicit expression for the fused covariance estimate. The convergence of this iterative algorithm is proved theoretically, and some good properties for the fused estimates are discussed.

The outline of this paper is organized as follows. In Section 2, the basic principles of information geometry and some useful results concerning the manifold of multivariate Gaussian distributions are introduced. Section 3 formulates a novel Lie algebraic fusion criterion and then develops the geodesic projection based algebraic fusion method. Simulation examples in Section 4 are provided to demonstrate the efficiency. Section 5 concludes.

### 1.1 Notations

Throughout this paper, all vectors are column vectors; Lightface letters denote scalars and scalar-valued mappings; Boldface lowercase letters denote vectors and tangent directions at a point; Boldface uppercase letters denote matrices and matrix-valued mappings; $R^{m}$ represents the set of all *m*-dimensional real vectors; $S_{+}^{m}$ is the set of *m* × *m* real symmetric and positive-definite matrices; The symbol **I**_*m*_ is the *m* × *m* identity matrix; tr(⋅) and (⋅)^*T*^ denote the trace and the transpose of a matrix, respectively; diag(⋅) means the diagonal matrix with a vector as its diagonal elements; Log(⋅) and EXP(·) are matrix logarithm and matrix exponential functions, respectively.

## 2 Preliminaries

### 2.1 Statistical manifold and fisher metric

Consider a family of probability densities

$$\begin{array}{c} S=\{p\left(x;\theta \right):\theta =\left(\theta^{1},\cdots ,\theta^{m}\right)\in \Theta \} \end{array}$$ (1)

with the global coordinate system ***θ*** = (*θ*^1^, …, *θ^m^*), where the parameter space Θ is an open set of $R^{m}$. For simplicity, we shall abbreviate the probability density *p*(***x***; ***θ***) in S as its coordinate ***θ***. The parameterized family S can be regarded as a Riemannian manifold by introducing the Fisher metric *g* derived from the *m* × *m* Fisher information matrix with

$$\begin{array}{c} g_{ij}\left(\theta \right)=E_{\theta }[\frac{\partial }{\partial \theta^{i}}\text{log}\;p\left(x;\theta \right)\frac{\partial }{\partial \theta^{j}}\text{log}\;p\left(x;\theta \right)] \end{array}$$ (2)

as the (*i*, *j*)-th entry, where $E_{\theta }\left[\cdot \right]$ is the mathematical expectation operator with respect to the PDF *p*(***x***; ***θ***).

Denote $T_{\theta }S$ as the vector space of tangent vectors at the point θ∈S, and the Fisher metric *g* can define an inner product on $T_{\theta }S$, written as

$$\begin{array}{c} {\left\langle \cdot ,\cdot \right\rangle }_{\theta }:T_{\theta }S\times T_{\theta }S\to R. \end{array}$$ (3)

Thus the norm of a tangent vector $\nu \in T_{\theta }S$ is given by ${∥\nu ∥}_{\theta }={\langle \nu ,\nu \rangle }_{\theta }^{1/2}$, and the induced geodesic distance called the Fisher information distance or the Rao distance is computed as follows:

$$\begin{array}{c} \ell_{F}\left(\theta_{0},\theta_{1}\right)≔\underset{\gamma \in \Gamma }{\text{min}\;}\left\{\ell \left(\gamma \right):\gamma \left(0\right)=\theta_{0},\gamma \left(1\right)=\theta_{1}\right\}, \end{array}$$ (4)

where the set Γ consists of all piecewise smooth curves connecting two endpoints ***θ***_0_ and ***θ***_1_, and the length of the geodesic segment *γ*(*t*), *t* ∈ [0, 1] is obtained by integrating the norm of its tangent vector $\dot{\theta }\left(t\right)$:

$$\begin{array}{c} \ell \left(\gamma \right)≔\int_{0}^{1}{∥\dot{\theta }\left(t\right)∥}_{\theta (t)}dt. \end{array}$$ (5)

Given any $\nu \in T_{\theta_{0}}S$, there exists a geodesic curve ***γ***(*t*; ***θ***_0_, ***ν***), *t* ∈ [0, 1] starting from ***θ***_0_ in the direction ***ν***. In differential geometry, the exponential mapping is defined as

$$\begin{array}{c} \begin{array}{cc} {\text{exp}\;}_{\theta_{0}}:T_{\theta_{0}}S & \to S\\ \nu & \mapsto {\text{exp}\;}_{\theta_{0}}\nu =\gamma \left(1;\theta_{0},\nu \right). \end{array} \end{array}$$ (6)

Conversely, denoting ***θ***_1_ = ***γ***(1; ***θ***_0_, ***ν***), the logarithmic mapping ${\text{log}\;}_{\theta_{0}}\left(\theta_{1}\right)$ is equal to the tangent vector ***ν*** at the initial point ***θ***_0_. The manifold retraction *R*(⋅), as the generalization of the exponential mapping, is essentially a smooth mapping from the tangent bundle $TS=\{T_{\theta }S:\theta \in \Theta \}$ into the manifold S (see, e.g., [23, 24]), and its inverse $R_{\theta }^{-1}\left(\cdot \right)$ is called the lifting mapping that exists in some neighborhood of ***θ***. In practice, the logarithmic and exponential mappings are the commonly used lifting and retraction mappings, respectively.

### 2.2 Information geometry of Gaussian distributions

An *m*-dimensional random vector **x** has the Gaussian distribution *N*(***μ***, **Σ**) with mean vector ***μ*** and covariance matrix $\Sigma \in S_{+}^{m}$. The Gaussian manifold

$$\begin{array}{c} N^{m}=\left\{N\left(\mu ,\Sigma \right):\mu \in R^{m},\Sigma \in S_{+}^{m}\right\} \end{array}$$ (7)

with Fisher metric and Levi–Civita connection, has the global coordinate system ***θ*** = (***μ***, **Σ**). For brevity, we shall abbreviate the Gaussian distribution *N*(***μ***, **Σ**) in $N^{m}$ to its coordinate ***θ*** = (***μ***, **Σ**) without confusion.

For the frequently discussed Gaussian submanifold

$$\begin{array}{c} N_{\mu }^{m}=\left\{N\left(\mu ,\Sigma \right):\Sigma \in S_{+}^{m}\right\} \end{array}$$ (8)

with a fixed mean vector $\mu \in R^{m}$, the geodesic projection distance from a given point ***θ***_0_ = (***μ***_0_, **Σ**_0_) in $N^{m}$ onto $N_{\mu }^{m}$ is defined as

$$\begin{array}{c} d\left(\theta_{0},N_{\mu }^{m}\right)≔\underset{\theta \in N_{\mu }^{m}}{\text{inf}\;}\ell_{F}\left(\theta_{0},\theta \right). \end{array}$$ (9)

Although the geodesic distance between two points in $N^{m}$ is not available in the general case with *m* > 1, the resulting geodesic projection ***θ***_*p*_ = (***μ***, **Σ**_*p*_) on $N_{\mu }^{m}$ has been derived in [22, 25] with an explicit expression

$$\begin{array}{c} \Sigma_{p}=\Sigma_{0}+\frac{1}{2}\left(\mu -\mu_{0}\right){\left(\mu -\mu_{0}\right)}^{T}. \end{array}$$ (10)

## 3 Lie algebraic estimation fusion

Consider a distributed dynamic system with *N* sensors observing a common state $x\in R^{m}$. As practical scenarios mentioned in [11], the dynamic system is assumed to have Gaussian process and observation noises. Denote the local PDF from the *k*-th sensor by $p_{k}=N\left({\hat{x}}_{k},P_{k}\right)$ with known local estimate $\left({\hat{x}}_{k},P_{k}\right)$, and the fused PDF by $\hat{p}=N\left(\hat{x},P\right)$.

According to Eq (10), we project each *p*_*k*_ along the orthogonal geodesic curve in $N^{m}$ onto the submanifold $N_{\mu }^{m}$ with ***μ*** = **x** to get the geodesic projection ${\overset{˘}{p}}_{k}=N\left(x,{\overset{˘}{P}}_{k}\left(x\right)\right)$, where

$$\begin{array}{c} {\overset{˘}{P}}_{k}\left(x\right)=P_{k}+\frac{1}{2}\left(x-{\hat{x}}_{k}\right){\left(x-{\hat{x}}_{k}\right)}^{T},\;k=1,\ldots ,N. \end{array}$$ (11)

Moreover, we replace each local posterior density *p*_*k*_ with its geodesic projection ${\overset{˘}{p}}_{k}$ to measure the dissimilarity between *p*_*k*_ and the sought-for fused posterior density *p* = *N*(**x**, **Σ**) with undetermined **x** and **Σ**.

### 3.1 Lie algebraic settings

Since $N_{\mu }^{m}$ inherits the topology and metric from $N^{m}$ and only uses **Σ** coordinate system [26], the inner product defined on $N_{\mu }^{m}$ is given as

$$\begin{array}{c} {\left\langle u_{1},u_{2}\right\rangle }_{g}=\frac{1}{2}\text{tr}(u_{1}G^{-1}u_{2}G^{-1}) \end{array}$$ (12)

for any fixed **g** = (**x**, **G**) in $N_{\mu }^{m}$ with ***μ*** = **x**, and two arbitrary vectors **u**_1_, **u**_2_ in the tangent space $T_{g}N_{\mu }^{m}$ at the point **g**. Then the norm of a tangent vector $u\in T_{g}N_{\mu }^{m}$ is defined as

$$\begin{array}{c} {∥u∥}_{g}={\left\langle u,u\right\rangle }_{g}^{\frac{1}{2}}. \end{array}$$ (13)

We can identify the symmetric positive-definite matrix space $S_{+}^{m}$ with the submanifold $N_{\mu }^{m}$ owing to the same Riemannian metric induced by Eq (12). Meanwhile, $S_{+}^{m}$ can even be regarded as a Lie group by introducing two operations from [27], which are compatible with the differential structure of $S_{+}^{m}$. One is the logarithmic multiplication ⊕ given by

$$\begin{array}{c} A⊕B≔\text{Exp}(\text{Log}(A)+\text{Log}(B)) \end{array}$$ (14)

for any $A,B\in S_{+}^{m}$, and the other is the logarithmic scalar multiplication ⊗ defined by

$$\begin{array}{c} \lambda ⊗A=\text{Exp}(\lambda \;\text{Log}(A)) \end{array}$$ (15)

for any λ∈R. Throughout the remainder, we denote $S_{+}^{m}$ as $S_{+}^{m}$ to stress its algebraic structure. For any $A\in S_{+}^{m}$, the left translation by **A**^−1^ is denoted as $L_{A^{-1}}\left(B\right)=A^{-1}⊕B$ for any $B\in S_{+}^{m}$. In Lie group theory [23], $L_{A^{-1}}\left(B\right)$ represents the difference between **A** and **B**.

The tangent space $T_{e}S_{+}^{m}$ at the identity element **e** = **I**_*m*_, also known as the Lie algebra g, is linearly isomorphic to the space $S^{m}$ of real symmetric matrices [28]. By respectively assimilating ⊕ and ⊗ to addition + and scalar multiplication × and identifying the vector space (g,+,×) with $\left(S^{m},+,\times \right)$, the map Log(⋅) constructs a linear isomorphism from $\left(S_{+}^{m},⊕,⊗\right)$ to (g,+,×), and is also a diffeomorphism from a neighborhood of e in $S_{+}^{m}$ to that of the null element **0** in g [29]. Also, *R*_**e**_(·) = Exp(·) and its inverse $R_{e}^{-1}\left(\cdot \right)=\text{Log}\left(\cdot \right)$ are the commonly used retraction and lifting maps of $S_{+}^{m}$ at **e**, respectively. Therefore, the displacement between **A** and **B** in $S_{+}^{m}$ can be evaluated by the vector $R_{e}^{-1}\left(L_{A^{-1}}\left(B\right)\right)=\text{Log}\left(B\right)-\text{Log}\left(A\right)$ in g.

### 3.2 Fusion criterion

For convenience, we denote *p* = *N*(**x**, **Σ**) and ${\overset{˘}{p}}_{k}=N\left(x,{\overset{˘}{P}}_{k}\left(x\right)\right)$ as their coordinates **Σ** and ${\overset{˘}{P}}_{k}\left(x\right)$, respectively. By fusing the geodesic projections ${\overset{˘}{p}}_{k},\;k=1,\ldots ,N$, the detailed operations of the Lie algebraic fusion to achieve the fused posterior density *p* are illustrated as follows:

(i) Move all geodesic projections ${\overset{˘}{P}}_{1}\left(x\right),\ldots ,{\overset{˘}{P}}_{N}\left(x\right)$ into a neighborhood of the identity element $e\in S_{+}^{m}$ via the left translation $L_{\Sigma^{-1}}\left(\cdot \right)$ to get $\Sigma^{-1}⊕{\overset{˘}{P}}_{k}\left(x\right)$, and then shift them into the Lie algebra g by the lifting map $R_{e}^{-1}\left(\cdot \right)$ to get
  $$\begin{array}{c} v_{k}=\text{Log}\left({\overset{˘}{P}}_{k}\left(x\right)\right)-\text{Log}\left(\Sigma \right)\in g,\;k=1,\ldots ,N. \end{array}$$ (16)
  Here, **v**_*k*_ measures the displacement between the fused posterior density *p* and the geodesic projection ${\overset{˘}{p}}_{k}$.
(ii) Motivated from the commonly adopted arithmetic mean as the barycenter of data points in Euclidean space, we can handle the estimation fusion with the Euclidean operation, i.e., the arithmetic averaging in the Log-Euclidean domain, due to the linear structure of g. Let $\bar{v}\left(x,\Sigma \right)$ measure the average displacement between the fused posterior density *p* and all geodesic projections ${\overset{˘}{p}}_{k}$:
  $$\begin{array}{c} \bar{v}\left(x,\Sigma \right)=\sum_{k=1}^{N}c_{k}v_{k}\in g. \end{array}$$ (17)
  Consider applying the real weight vector **c** = [*c*_1_,…,*c_N_*]^*T*^ to minimize the sum of squared norm distances from the average vector $\sum_{k=1}^{N}c_{k}v_{k}$ to *N* vectors **v**_*k*_:
  $$\begin{array}{c} \underset{c\in R^{N}}{\text{arg}\;\text{min}\;}\sum_{k=1}^{N}{∥v_{k}-\bar{v}\left(x,\Sigma \right)∥}_{e}^{2}. \end{array}$$ (18)
  This is a convex quadratic programming problem. Setting partial derivatives of the objective function of Eq (18) with respect to *c*_*l*_ to zeros, i.e.,
  $$\begin{array}{c} \sum_{k=1}^{N}c_{k}{\left\langle v_{k},v_{l}\right\rangle }_{e}={\left\langle \bar{v},v_{l}\right\rangle }_{e},\;l=1,\ldots ,N, \end{array}$$ (19)
  we obtain the optimal weight coefficients
  $$\begin{array}{c} c_{k}=\frac{1}{N},\;k=1,\ldots ,N. \end{array}$$ (20)
  and then from Eqs (16), (17) and (20),
  $$\begin{array}{c} \bar{v}\left(x,\Sigma \right)=\frac{1}{N}\sum_{k=1}^{N}\text{Log}\left({\overset{˘}{P}}_{k}\left(x\right)\right)-\text{Log}\left(\Sigma \right). \end{array}$$ (21)
(iii) Using the Euclidean structure on the Lie algebra g, we take the norm of the average displacement vector $\bar{v}\left(x,\Sigma \right)$ as the cost, and by minimizing the cost we formulate a novel algebraic fusion criterion as
  $$\begin{array}{c} \left(\hat{x},P\right)=\underset{\left(x,\Sigma \right)\in R^{m}\times S_{+}^{m}}{\text{arg}\;\text{min}\;}{∥\bar{v}\left(x,\Sigma \right)∥}_{e}^{2}. \end{array}$$ (22)

### 3.3 Fusion algorithm

The optimization of Eq (22) can be further decomposed into two steps—first over **x** and then over **Σ**:

$$\begin{array}{c} \left(\hat{x},P\right)=\underset{\Sigma \in S_{+}^{m}}{\text{arg}\;\text{min}\;}\underset{x\in R^{m}}{\text{min}\;}{∥\bar{v}\left(x,\Sigma \right)∥}_{e}^{2}. \end{array}$$ (23)

First, seek the fused mean $\hat{x}$ to minimize the norm of the average vector $\bar{v}\left(x,\Sigma \right)$ for any **Σ**:

$$\begin{array}{c} \hat{x}=\underset{x\in R^{m}}{\text{arg}\;\text{min}\;}\text{tr}({\left(\bar{v}\left(x,\Sigma \right)\right)}^{2}); \end{array}$$ (24)

Second, for $x=\hat{x}$, seek the fused covariance estimate **P** to minimize the norm of $\bar{v}\left(\hat{x},\Sigma \right)$:

$$\begin{array}{c} P=\underset{\Sigma \in S_{+}^{m}}{\text{arg}\;\text{min}\;}\text{tr}({\left(\bar{v}\left(\hat{x},\Sigma \right)\right)}^{2}). \end{array}$$ (25)

**Theorem 1**. *The fused mean estimate* $\hat{x}$ *in* Eq (24) *satisfies an implicit expression*

$$\begin{array}{c} \hat{x}={\left(\sum_{k=1}^{N}w_{k}\left(\hat{x}\right)P_{k}^{-1}\right)}^{-1}\sum_{k=1}^{N}w_{k}\left(\hat{x}\right)P_{k}^{-1}{\hat{x}}_{k} \end{array}$$ (26)

*with the weights*

$$\begin{array}{c} w_{k}\left(\hat{x}\right)=\frac{1}{1+\frac{1}{2}{\left(\hat{x}-{\hat{x}}_{k}\right)}^{T}P_{k}^{-1}\left(\hat{x}-{\hat{x}}_{k}\right)},\;k=1,\ldots ,N, \end{array}$$ (27)

*and the fused covariance* **P** *in* Eq (25) *is*

$$\begin{array}{c} P=\text{Exp}(\frac{1}{N}\sum_{k=1}^{N}\text{Log}({\overset{˘}{P}}_{k}\left(\hat{x}\right))). \end{array}$$ (28)

*Proof*. Denoting a function of variable **x** as

$$\begin{array}{c} \psi_{\Sigma }\left(x\right)=\text{tr}({\left(\bar{v}\left(x,\Sigma \right)\right)}^{2})=\text{tr}({\left(\frac{1}{N}\sum_{k=1}^{N}\text{Log}\left({\overset{˘}{P}}_{k}\left(x\right)\right)-\text{Log}\left(\Sigma \right)\right)}^{2}) \end{array}$$ (29)

for any given **Σ**, we can easily obtain the derivative

$$\begin{array}{c} \frac{d\psi_{\Sigma }\left(x\right)}{dx}=\frac{2}{N}\bar{v}\left(x,\Sigma \right)\cdot \sum_{k=1}^{N}{\overset{˘}{P}}_{k}{\left(x\right)}^{-1}\left(x-{\hat{x}}_{k}\right). \end{array}$$ (30)

The fused mean estimate $\hat{x}$ in Eq (24) satisfies d *ψ*_**Σ**_(**x**)/d **x** = 0. Considering the arbitrariness of variable **Σ** in Eq (24), we let $\hat{x}$ satisfy the stationary condition

$$\begin{array}{c} \sum_{k=1}^{N}{\overset{˘}{P}}_{k}{\left(\hat{x}\right)}^{-1}\left(\hat{x}-{\hat{x}}_{k}\right)=0. \end{array}$$ (31)

Inserting Sherman–Morrison formula in [30] into Eq (11), we have

$$\begin{array}{c} {\overset{˘}{P}}_{k}{\left(\hat{x}\right)}^{-1}=P_{k}^{-1}-\frac{P_{k}^{-1}\left(\hat{x}-{\hat{x}}_{k}\right){\left(\hat{x}-{\hat{x}}_{k}\right)}^{T}P_{k}^{-1}}{2+{\left(\hat{x}-{\hat{x}}_{k}\right)}^{T}P_{k}^{-1}\left(\hat{x}-{\hat{x}}_{k}\right)}. \end{array}$$ (32)

Then, substituting Eq (32) into Eq (31) yields

$$\begin{array}{c} \sum_{k=1}^{N}\frac{P_{k}^{-1}\left(\hat{x}-{\hat{x}}_{k}\right)}{1+\frac{1}{2}{\left(\hat{x}-{\hat{x}}_{k}\right)}^{T}P_{k}^{-1}\left(\hat{x}-{\hat{x}}_{k}\right)}=0, \end{array}$$ (33)

and thus Eq (26) follows from Eq (33).

Owing to the property of the function tr(⋅), the fused covariance (28) for the minimization problem in Eq (25) is obvious.

**Remark 1**. *As an arithmetic mean in the domain of matrix logarithms, the Log-Euclidean mean as shown in* Eq (28) *has been successfully applied in many areas such as elasticity theory* [31] *and image processing* [32–34].

In Theorem 1, the objective function in Eq (22) reaches its minimum value 0 through the fused estimate $\left(\hat{x},P\right)$. Moreover, due to the term ${\left(x-{\hat{x}}_{k}\right)}^{T}P_{k}^{-1}\left(x-{\hat{x}}_{k}\right)$ in Eq (27), the Lie fusion for $\hat{x}$ in Eq (26) is deemed robust and reliable against outliers. To further solve the implicit Eq (26) for the fused mean estimate $\hat{x}$, the following theorem provides a rigorous proof about the convergence of the fixed point iteration for $\hat{x}$.

**Theorem 2**. *By adopting the fixed point iteration*

$$\begin{array}{c} \xi_{t+1}={\left(\sum_{k=1}^{N}w_{k}\left(\xi_{t}\right)P_{k}^{-1}\right)}^{-1}\sum_{k=1}^{N}w_{k}\left(\xi_{t}\right)P_{k}^{-1}{\hat{x}}_{k} \end{array}$$ (34)

*with*

$$\begin{array}{c} w_{k}\left(\xi_{t}\right)=\frac{1}{1+\frac{1}{2}{\left(\xi_{t}-{\hat{x}}_{k}\right)}^{T}P_{k}^{-1}\left(\xi_{t}-{\hat{x}}_{k}\right)} \end{array}$$ (35)

*for k* = 1, …, *N*, *the iterative sequence* $\left\{\xi_{t},t\in N\right\}$ *is bounded and its accumulation point* $\hat{x}$ *as the final fused mean estimate satisfies the stationary condition shown in* Eq (33).

*Proof*. Denote two functions of variable $\xi \in R^{m}$ as

$$\begin{array}{cc} U(\xi ,\xi_{t}) & =-\sum_{k=1}^{N}\text{log}\;\left(w_{k}\left(\xi_{t}\right)\right)+\sum_{k=1}^{N}\frac{w_{k}\left(\xi_{t}\right)}{w_{k}\left(\xi \right)}, \end{array}$$ (36)

$$\begin{array}{c} \phi (\xi )=N-\sum_{k=1}^{N}\text{log}\;\left(w_{k}\left(\xi \right)\right). \end{array}$$ (37)

It is easily verified from Eqs (36) and (37) that

$$\begin{array}{c} \phi \left(\xi \right)\leq U(\xi ,\xi_{t},\;\phi \left(\xi_{t}\right)=U\left(\xi_{t},\xi_{t}\right). \end{array}$$ (38)

Define the map

$$\begin{array}{c} T\left(\xi_{t}\right)≔\xi_{t+1}=\underset{\xi \in R^{m}}{\text{arg}\;\text{min}\;}U\left(\xi ,\xi_{t}\right). \end{array}$$ (39)

Since *U*(***ξ***, ***ξ***_*t*_) is convex as a function of ***ξ***, the minimizer ***ξ***_*t*+1_ in Eq (39) is unique. Further setting the gradient of *U*(***ξ***, ***ξ***_*t*_) with respect to ***ξ*** to zero, we have

$$\begin{array}{c} T\left(\xi_{t}\right)={\left(\sum_{k=1}^{N}w_{k}\left(\xi_{t}\right)P_{k}^{-1}\right)}^{-1}\sum_{k=1}^{N}w_{k}\left(\xi_{t}\right)P_{k}^{-1}{\hat{x}}_{k}. \end{array}$$ (40)

Meanwhile, combining *ϕ*(***ξ***) ≥ *N* due to Eq (37), and the fact

$$\begin{array}{c} \phi \left(T\left(\xi_{t}\right)\right)=\phi \left(\xi_{t+1}\right)\leq U\left(\xi_{t+1},\xi_{t}\right)\leq \phi \left(\xi_{t}\right), \end{array}$$ (41)

we know that *ϕ*(***ξ***_*t*_) is decreasing and converges as *t* tends to + ∞, so the sets $\left\{\phi \left(\xi_{t}\right),\;t\in N\right\}$ and $\left\{\xi_{t},\;t\in N\right\}$ are bounded. Moreover, for any subsequence $\left\{\xi_{t_{i}},\;i\in N\right\}$ converging to $\hat{x}$, applying the continuity of maps *ϕ*(⋅) and *T*(⋅) leads to $\phi \left(T\left(\hat{x}\right)\right)=\phi \left(\hat{x}\right)$. As a result, it follows from the second inequality in Eq (41) that $U\left(T\left(\hat{x}\right),\hat{x}\right)=U\left(\hat{x},\hat{x}\right)$, and then $\hat{x}$ is the unique minimizer of $U(\xi ,\hat{x})$, i.e., $T\left(\hat{x}\right)=\hat{x}$, by the definition of *T*(⋅) in Eq (39). The theorem thus follows.

Note that the two fused estimates as shown in Eqs (26) and (28) satisfy the following desirable properties:

(i) Invariance under any affine transformation **Q**. If $\hat{x}$ is the fused estimate of ${\left\{{\hat{x}}_{k}\right\}}_{1\leq k\leq N}$, $Q\hat{x}$ is the fused estimate of ${\left\{Q{\hat{x}}_{k}\right\}}_{1\leq k\leq N}$. Also, if **Q** is an orthogonal matrix and **P** is the fused estimate of ${\left\{{\overset{˘}{P}}_{k}\right\}}_{1\leq k\leq N}$, **QPQ**^*T*^ is the fused estimate of ${\left\{Q{\overset{˘}{P}}_{k}Q^{T}\right\}}_{1\leq k\leq N}$.
(ii) Invariance under the inversion. If **P** is the fused estimate of ${\left\{{\overset{˘}{P}}_{k}\right\}}_{1\leq k\leq N}$, **P**^−1^ is the fused estimate of ${\left\{{\overset{˘}{P}}_{k}^{-1}\right\}}_{1\leq k\leq N}$.

In summary, combining the fixed point iteration (34) for $\hat{x}$ with the explicit expression (28) for the fused covariance estimate **P**, we outline the above fusion method in Algorithm 1 and call it the algebraic (GPA) fusion method based on geodesic projection.

**Remark 2**. *Many existing fusion methods, including the DCI, WDCI, FCI, KLA and GP, have identical forms as the CI, but in general with different weights. Especially when two sensors are used to track a one-dimensional target, the fused estimates for these fusion methods theoretically lie on the Euclidean line segment between two local estimates from sensors, whereas according to* Eqs (26) *and* (28), *the GPA does not. As shown in* [19], *the Gaussian Riemannian manifold* $N^{m}$ *is not flat, so the proposed GPA is more reasonable owing to its utilizing the geometric and algebraic structures on* $N^{m}$. *In Section 4.1, we take the two-sensor case for instance to validate the above fact*.

**Algorithm 1:** GPA Distributed Fusion Algorithm.

**Input**: ${\left\{({\hat{x}}_{k},P_{k})\right\}}_{k=1,\ldots ,N}$ and tolerances *ϵ*_1_, *ϵ*_2_

**Output**: $\left(\hat{x},P\right)$

**1** Set *t* = 0, *r*_1_ = *ϵ*_1_ and *r*_2_ = *ϵ*_2_;

**2** Initiate ***ξ***_0_ using the CI state estimate;

**3** **while** *r*_1_ ≥ *ϵ*_1_ *and r*_2_ ≥ *ϵ*_2_ **do**

**4**  Run mean iteration shown in Eq (34);

**5**  Compute the Frobenius norm *r*_1_ = ∥***ξ***_*t*+1_ − ***ξ***_*t*_∥_*F*_;

**6**  Compute by Eq (33) the Frobenius norm $r_{2}={∥\sum_{k=1}^{N}w_{k}\left(\xi_{t+1}\right)P_{k}^{-1}\left(\xi_{t+1}-{\hat{x}}_{k}\right))∥}_{F}$;

**7**  Set *t* ← *t* + 1

**8 end**

**9** $\hat{x}=\xi_{t}$ ;

**10** Compute ${\overset{˘}{P}}_{k}\left(\hat{x}\right)$ by inserting $x=\hat{x}$ into Eq (11);

**11** Compute the covariance estimate **P** using Eq (28);

**12 return** $\left(\hat{x},P\right)$ .

**Remark 3**. *An extension of the GPA method to non-Gaussian PDFs is far more difficult. For the Riemannian manifold composed of multivariate non-Gaussian distributions, it is a great challenge to obtain the explicit geodesic projection, which is the key requirement for our fusion method*.

## 4 Simulations

In this section, three numerical examples (i.e., one-dimensional static target, and linear and nonlinear dynamic systems) are provided to demonstrate the performance of the proposed GPA method in distributed estimation fusion. All fusion algorithms are implemented in Octave on a computer with Intel Core i7-10870H 2.20 GHz processor and the corresponding program codes are accessible via the doi “10.6084/m9.figshare.25116515”.

### 4.1 One-dimensional static target

**Fig 1:**
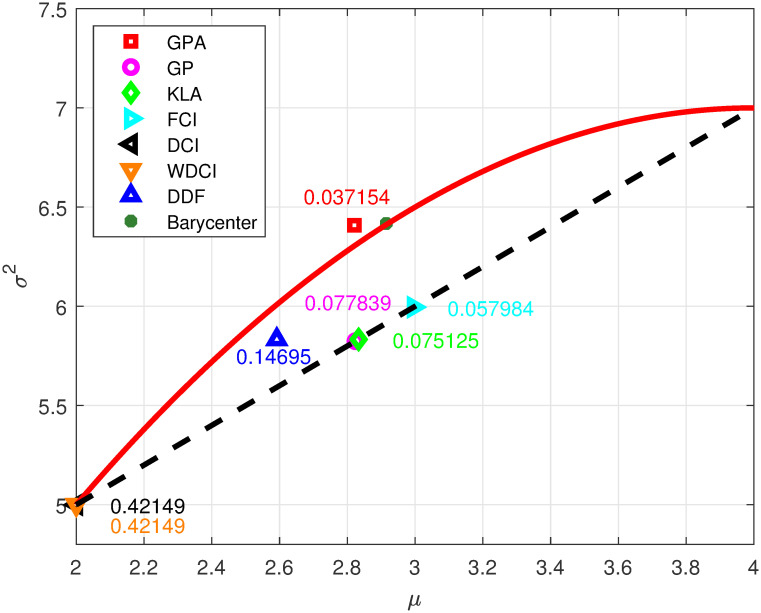
The figure illustrates the geodesic distances (labeled by the corresponding marks) between the informative barycenter and all the fused densities. The solid red curve represents the geodesic curve linking two local densities.

To intuitively compare the performance difference of these fusion methods, we use two local estimates $\left({\hat{x}}_{1},P_{1}\right)=\left(2,5\right)$ and $\left({\hat{x}}_{2},P_{2}\right)=\left(4,7\right)$ from two sensors for fusion. As defined in [22], the informative barycenter is an optimal point on the geodesic segment by minimizing the sum of its squared geodesic distances to two endpoints $\left({\hat{x}}_{1},P_{1}\right)$ and $\left({\hat{x}}_{2},P_{2}\right)$. Fig 1 clearly displays all geodesic distances between the informative barycenter and the fused densities. As stated in Remark 2, the fused estimates for the DCI, FCI, KLA, GP and WDCI lie on the (straight) Euclidean segment between $\left({\hat{x}}_{1},P_{1}\right)$ and $\left({\hat{x}}_{2},P_{2}\right)$, while the GPA and the DDF do not. Moreover, compared to other methods, we can observe that the estimate of GPA is closest to the barycenter. Therefore, the GPA is considered more reasonable because the Gaussian manifold is indeed non-Euclidean.

### 4.2 Linear dynamic system

Consider the following dynamic system with one fusion center and *N* sensors for tracking a two-dimensional target:

$$\begin{array}{c} x_{k+1}=[\begin{array}{cc} 1 & T\\ 0 & 1 \end{array}]x_{k}+[\begin{array}{cc} \frac{T^{2}}{2} & 0\\ 0 & 1 \end{array}]\omega_{k}, \end{array}$$ (42)

$$\begin{array}{c} z_{k}^{i}=H_{k}^{i}x_{k}+v_{k}^{i},\;i=1,2, \end{array}$$ (43)

$$\begin{array}{c} z_{k}^{j}=H_{k}^{j}x_{k}+v_{k}^{j},j=3,\ldots ,N, \end{array}$$ (44)

where the sampling period *T* = 1s, the state **x**_*k*_ has two components (i.e., the position and the velocity), $H_{k}^{1}=\left[1,0\right]$, $H_{k}^{2}=\left[0,1\right]$ and $H_{k}^{j}=\text{diag}\left(\left[1,j-2\right]\right)$ for *j* = 3, …, *N*. The joint observation noise $v_{k}={\left[v_{k}^{1},v_{k}^{2},{\left(v_{k}^{3}\right)}^{T},\ldots ,{\left(v_{k}^{N}\right)}^{T}\right]}^{T}$ follows *N*(**0**, **R**_*k*_) and its covariance **R**_*k*_ is a positive-definite Toeplitz matrix with main diagonal $\sigma^{2}1_{2N-2}$ and two sub-diagonals $\rho \sigma^{2}1_{2N-3}$, where **1**_*n*_ is an *n*-dimensional vector with all entries 1 and −1/(2 cos(*π*/(2*N − 1))) < *ρ* < 1/(2 cos(*π*/(2*N − 1))) ensures that **R**_*k*_ is positive-definite. Moreover, the process noise ***ω***_*k*_ follows ***N***(**0**, **Q**_*k*_ with **Q**_*k*_ = *γ* · diag([12, 4]), the initial state **x**_0_ is generated by $N(\hat{x}\left(0|0\right),P\left(0|0\right))$ with $\hat{x}\left(0|0\right)={\left[10,5\right]}^{T}$ and **P**(0|0) = diag([100, 25]), and ***ω***_*k*_ and **v**_*k*_ are mutually uncorrelated at each instant *k*.

The local estimate $\left({\hat{x}}_{i}\left(k|k\right),P_{i}\left(k|k\right)\right)$ is calculated by the standard Kalman filter, and then propagated synchronously to the fusion center. Further, the fusion center applies a specified fusion method to obtain the fused estimate $\left(\hat{x}\left(k|k\right),P\left(k|k\right)\right)$, and transmits it back to the local sensors. In order to show the performance of various fusion algorithms, i.e., the DCI, WDCI, FCI, KLA, DDF, GP and GPA, with different cross-correlations among local estimation errors, different number of sensors, different measurement matrices, and different **Q**_*k*_ and **R**_*k*_, we respectively vary *ρ*, *γ*, *N* and *σ*^2^, and compare the averaged root mean squared errors (ARMSEs) of the position and the velocity over 100 time steps and 500 Monte Carlo runs. Specifically, four different cases are listed as follows:

- Case I: Fix *γ* = 1, *N* = 3 and *σ*^2^ = 10, and the correlation coefficient *ρ* varies from −0.6 to 0.6 with step 0.1.
- Case II: Fix *ρ* = 0.1, *N* = 3 and *σ*^2^ = 10, and *γ* varies from 1 to 6 with step 1.
- Case III: Fix *ρ* = 0.1, *γ* = 1 and *σ*^2^ = 10, and the number *N* of sensors varies from 2 to 7.
- Case IV: Fix *ρ* = 0.1, *γ* = 1 and *N* = 3, and *σ*^2^ varies from 5 to 30 with step 5.

**Fig 2:**
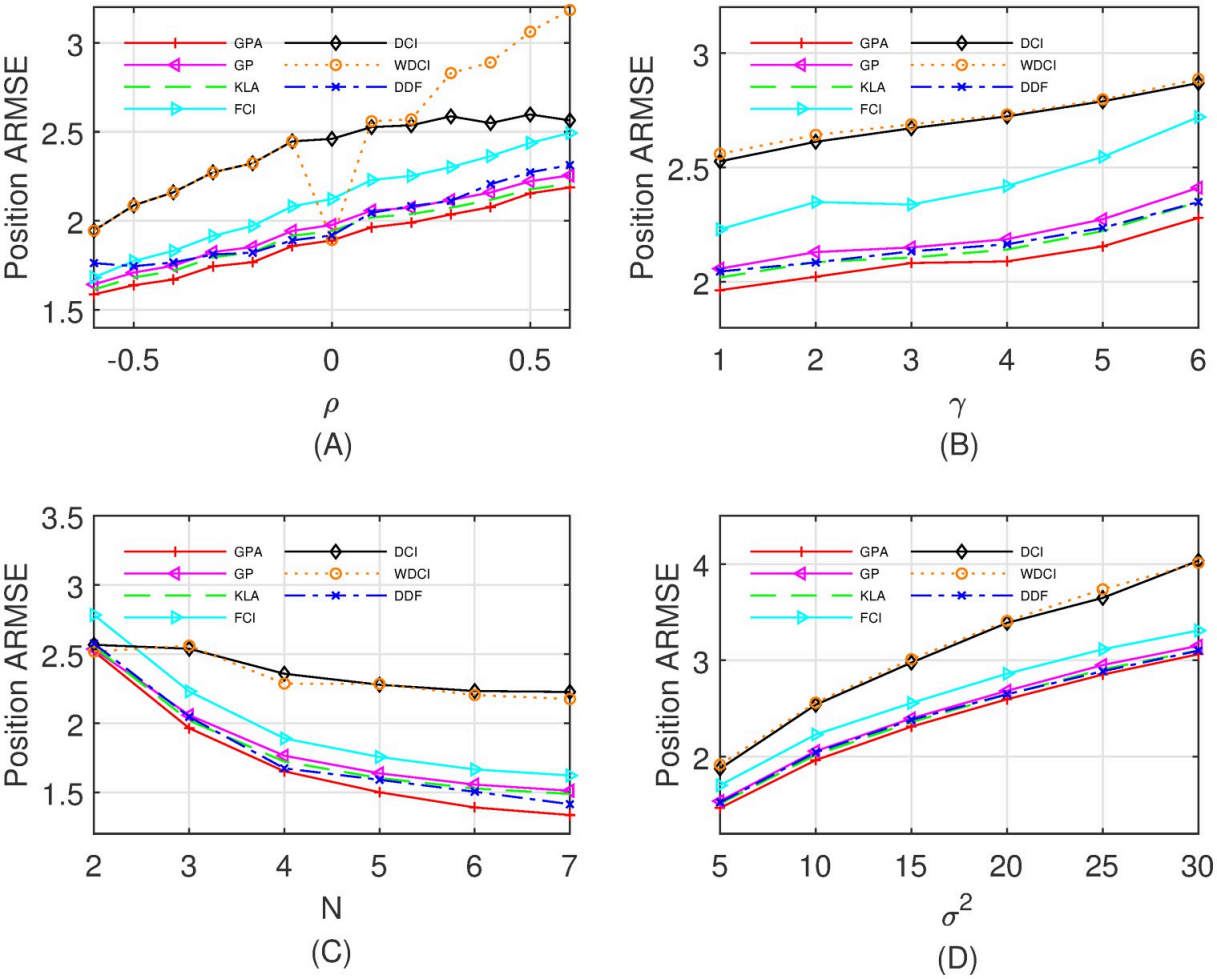
The ARMSEs of fused results for position estimates. (A) Case I with *ρ* ∈ [−0.6, 0.6]; (B) Case II with *γ* ∈ {1, 2, …, 6}; (C) Case III with *N* ∈ {2, 3, …, 7}; (D) Case IV with *σ*^2^ ∈ {5, 10, …, 30}.

**Fig 3:**
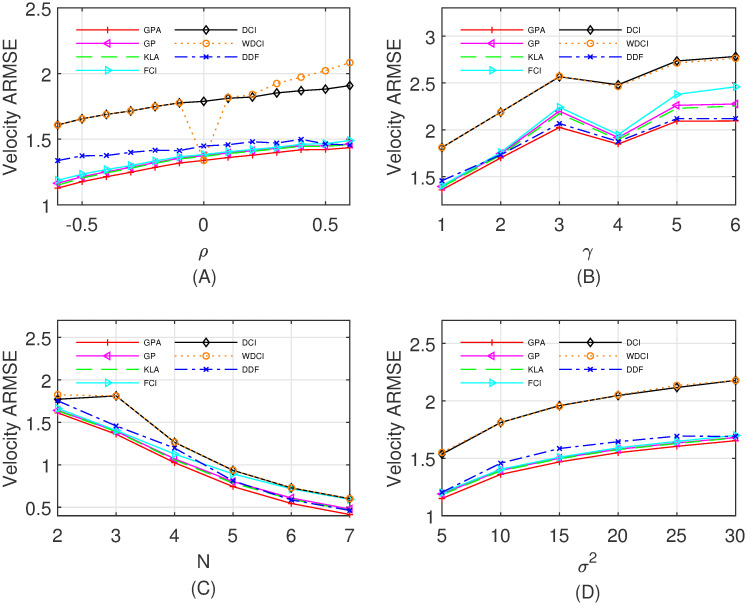
The ARMSEs of fused results for velocity estimates. (A) Case I with *ρ* ∈ [−0.6, 0.6]; (B) Case II with *γ* ∈ {1, 2, …, 6}; (C) Case III with *N* ∈ {2, 3, …, 7}; (D)Case IV with *σ*^2^ ∈ {5, 10, …, 30}.

As shown in Figs 2 and 3(A)–3(D) illustrate the fusion performance of all compared fusion methods as the correlation coefficient, the process noise, the number of sensors and the measurement noise increase, respectively. It is evident that the proposed fusion algorithm GPA is consistently better for the ARMSEs of the position and the velocity than the other fusion methods in four different cases. We contribute it to utilizing the geometric and algebraic structures on the Gaussian manifold to fuse local estimates. Note that the WDCI in Figs 2(A) and 3(A) has a very close performance as the GPA only if each pair of local estimates is not correlated (i.e., *ρ* = 0), and otherwise performs poorly. Also, the total (100 steps, 500 Monte Carlo runs, *ρ* = 0.1) computation time of the compared fusion algorithms in Case I is reported in Table 1, which demonstrates that the proposed GPA has a low computation cost.

**Table 1:** Computation time (Seconds).

Fusion Method	KLA	FCI	GPA	GP	DCI	WDCI	DDF
Time	3.19	8.82	13.66	21.80	63.80	118.08	4416.72

### 4.3 Nonlinear dynamic system

As [35], we consider a two-dimensional dynamic system with the linear motion model

$$\begin{array}{c} x_{k+1}=F_{k}x_{k}+\omega_{k}, \end{array}$$ (45)

and the nonlinear observation model

$$\begin{array}{c} z_{k}^{i}=\sqrt{x_{k}^{2}+y_{k}^{2}}+v_{k}^{i},\;i=1,2,\ldots ,N, \end{array}$$ (46)

where **F**_*k*_ = diag([1, 1]), and the state **x**_*k*_ = [*x_k_*, *y_k_*]^*T*^ represents the position of the target moving in the *xy*-plane. The joint observation noise $v_{k}={\left[v_{k}^{1},v_{k}^{2},\ldots ,v_{k}^{N}\right]}^{T}$ follows *N*(**0**, **R**_*k*_) and the Toeplitz matrix **R**_*k*_ has the main diagonal *σ*^2^**1**_*N*_ and two sub-diagonals *ρσ*^2^**1**_*N*−1_. Also, the process noise ***ω***_*k*_ follows the Gaussian distribution ***N***(**0**, **Q**_*k*_) with **Q**_*k*_ = *γ* · diag([2, 1]), the initial state **x**_0_ is generated from $N(\hat{x}\left(0|0\right),P\left(0|0\right))$ with $\hat{x}\left(0|0\right)={\left[10,5\right]}^{T}$ and **P**(0|0) = diag([16, 9]), and ***ω***_*k*_ and **v**_*k*_ are mutually uncorrelated at each instant *k*.

The local estimate $\left({\hat{x}}_{i}\left(k|k\right),P_{i}\left(k|k\right)\right)$ is calculated by the unscented filter [36], and then propagated synchronously to the fusion center. As in the linear Gaussian system (42)–(44), we respectively vary *ρ*, *γ*, *N* and *σ*^2^, and compare the ARMSEs of the state estimate over 100 time steps and 500 Monte Carlo runs. Specifically, four different cases are listed as follows:

- Case I: Fix *γ* = 1, *N* = 3 and *σ*^2^ = 4, and the correlation coefficient *ρ* varies from −0.7 to 0.7 with step 0.1.
- Case II: Fix *ρ* = −0.1, *N* = 3 and *σ*^2^ = 4, and *γ* varies from 1 to 6 with step 1.
- Case III: Fix *ρ* = −0.1, *γ* = 1 and *σ*^2^ = 4, and the number *N* of sensors varies from 2 to 7.
- Case IV: Fix *ρ* = −0.1, *γ* = 1 and *N* = 3, and *σ*^2^ varies from 2 to 12 with step 2.

**Fig 4:**
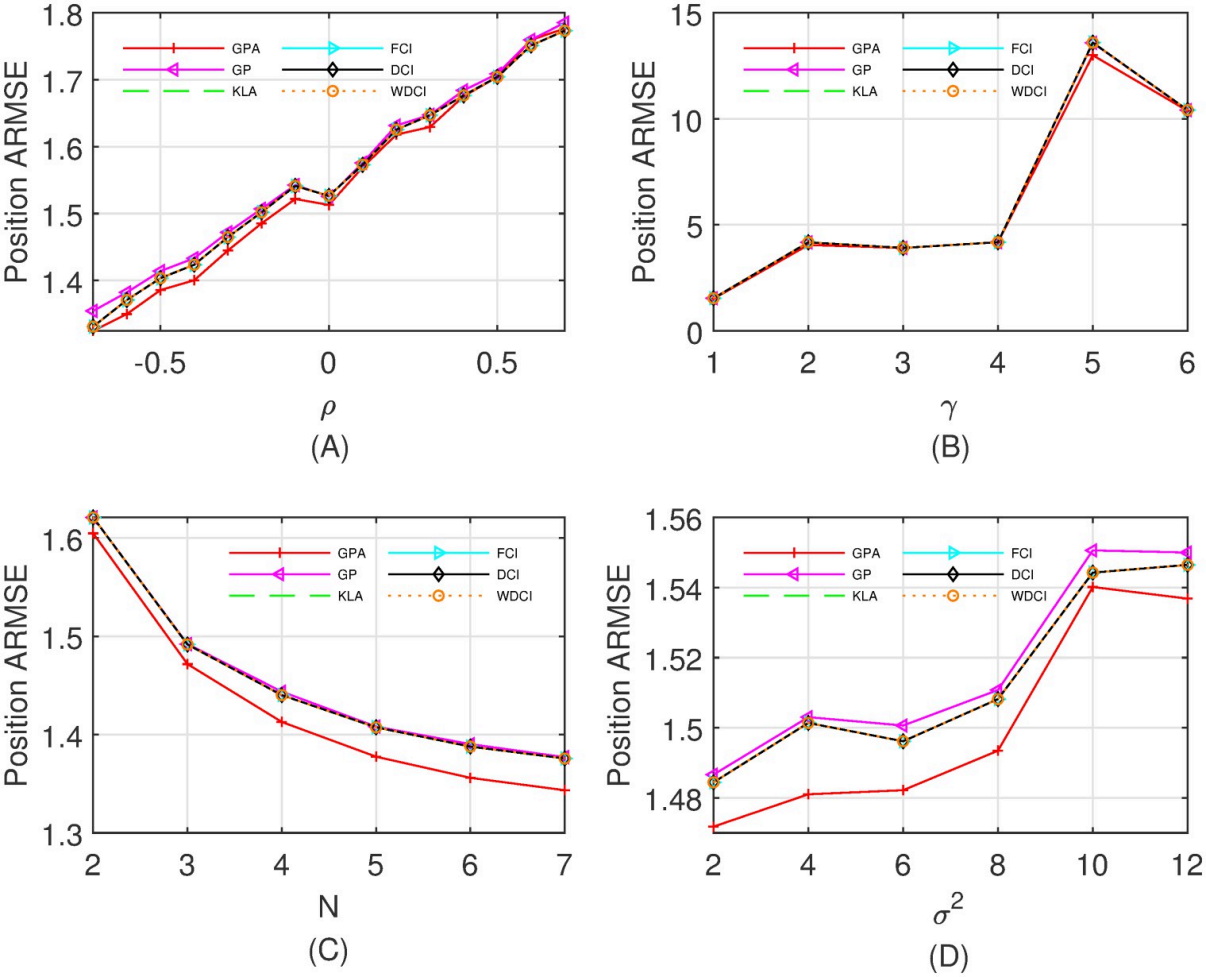
The ARMSEs of fused results for position estimates. (A) Case I with *ρ* ∈ [−0.7, 0.7]; (B) Case II with *γ* ∈ {1, 2, …, 6}; (C) Case III with *N* ∈ {2, 3, …, 7}; (D) Case IV with *σ*^2^ ∈ {2, 4, …, 12}.

From the comparisons of the ARMSEs of position estimates in Fig 4, we can find that the proposed fusion algorithm GPA is consistently better than the other fusion methods in four different cases, and the KLA, FCI, DCI, and WDCI have nearly the same performance. Moreover, Table 2 reports the total (100 steps, 500 Monte Carlo runs, *ρ* = −0.1) computation time of all compared fusion algorithms in Case I, indicating the low computation cost of the GPA. It is worth noting that the DDF is not shown in Fig 4 owing to its very poor performance and high computation cost.

**Table 2:** Computation time (Seconds).

Fusion Method	KLA	FCI	GPA	GP	DCI	WDCI
Time	4.60	10.78	12.36	18.43	44.34	70.31

### 4.4 Vehicle dynamic model

**Fig 5:**
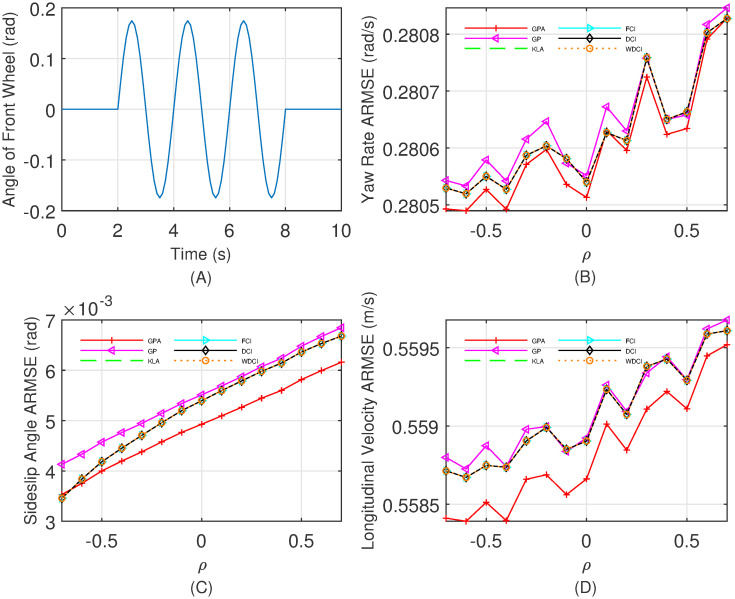
The change of front-wheel angle for (A) and the ARMSEs of fused results vs. *ρ* ∈ [−0.7, 0.7] for (B) the estimates of yaw velocity; (C) the estimates of sideslip angle; (D) the estimates of longitudinal velocity.

Consider a three-degree-of-freedom (TDF) model (see, e.g., [37]) to describe the coupled dynamics characteristics of the proceeding vehicle with one fusion center and *N* sensors for tracking this vehicle, whose state equations can be formulated as

$$\begin{array}{c} \dot{r}=\frac{a^{2}k_{1}+b^{2}k_{2}}{I_{z}v_{x}}r+\frac{ak_{1}-bk_{2}}{I_{z}}\beta -\frac{ak_{1}}{I_{z}}\delta , \end{array}$$ (47)

$$\begin{array}{c} \dot{\beta }=(\frac{ak_{1}-bk_{2}}{mv_{x}^{2}}-1)r+\frac{k_{1}+k_{2}}{mv_{x}}\beta -\frac{k_{1}}{mv_{x}}\delta , \end{array}$$ (48)

$$\begin{array}{c} {\dot{v}}_{x}=r\beta v_{x}+a_{x}, \end{array}$$ (49)

and measurement equation as

$$\begin{array}{c} a_{y}^{i}=\frac{ak_{1}-bk_{2}}{mv_{x}}r+\frac{k_{1}+k_{2}}{m}\beta -\frac{k_{1}}{m}\delta ,\;i=1,2,\ldots ,N, \end{array}$$ (50)

where the three dimensional state vector consists of the yaw rate *r*, the sideslip angle *β*, and the longitudinal velocity *v*_*x*_, and the lateral acceleration *a*_*y*_ is the measurement output. The simulation test environment in CarSim 2016 is set to a typical sinusoidal steering condition with a friction coefficient of 0.85, the lateral acceleration *a*_*x*_ is from the CarSim output, and the curve of the front-wheel steering angle *δ* is depicted in Fig 5(A) with a amplitude of 0.1744 rad and a period of 2 s. Other parameters in this model are listed as follows: the distance from the center of gravity to the front axle *a* = 1.066 m, the distance from the center of gravity to the rear axle *b* = 1.544 m, the vehicle mass *m* = 1458.4 kg, the cornering stiffness of the front axle *k*_1_ = −10^5^ N/rad, the cornering stiffness to the rear axle *k*_2_ = −11 × 10^4^ N/rad, the moment *I*_*z*_ = 2768 kg ⋅ m^2^ of inertia around the *Z* axis.

To estimate the vehicle state vector, the TDF model can be transformed into the following discrete-time state-space model

$$\begin{array}{c} {}[\begin{array}{c} r_{k+1}\\ \beta_{k+1}\\ {\left(v_{x}\right)}_{k+1} \end{array}]=[\begin{array}{c} r_{k}+(\frac{a^{2}k_{1}+b^{2}k_{2}}{I_{z}{\left(v_{x}\right)}_{k}}r_{k}+\frac{ak_{1}-bk_{2}}{I_{z}}\beta_{k}-\frac{ak_{1}}{I_{z}}\delta_{k})\Delta t\\ \beta_{k}+((\frac{ak_{1}-bk_{2}}{m{\left(v_{x}\right)}_{k}^{2}}-1)r_{k}+\frac{k_{1}+k_{2}}{m{\left(v_{x}\right)}_{k}}\beta_{k}-\frac{k_{1}}{m{\left(v_{x}\right)}_{k}}\delta_{k})\Delta t\\ {\left(v_{x}\right)}_{k}+(r_{k}\beta_{k}{\left(v_{x}\right)}_{k}+{\left(a_{x}\right)}_{k})\Delta t \end{array}]+\omega_{k}, \end{array}$$ (51)

$$\begin{array}{c} {\left(a_{y}\right)}_{k}^{i}=\frac{ak_{1}-bk_{2}}{m{\left(v_{x}\right)}_{k}}r_{k}+\frac{k_{1}+k_{2}}{m}\beta_{k}-\frac{k_{1}}{m}\delta_{k}+\nu_{k}^{i},\;i=1,2,\ldots ,N, \end{array}$$ (52)

where the sampling period Δ*t* = 0.02*s*. The joint observation noise $\nu_{k}={\left[\nu_{k}^{1},\nu_{k}^{2},\ldots ,\nu_{k}^{N}\right]}^{T}$ follows the Gaussian distribution *N*(**0**, **R**_*k*_) and the Toeplitz matrix **R**_*k*_ has the main diagonal **1**_*N*_ and two sub-diagonals *σ***1**_*N*−1_. Also, the process noise ***ω***_*k*_ follows ***N***(**0**, **Q**_*k*_) with **Q**_*k*_ = diag([*π*/180, *π*/180, 0.1]), the initial state **x**_0_ is generated from $N(\hat{x}\left(0|0\right),P\left(0|0\right))$ with $\hat{x}\left(0|0\right)={\left[0,0,25\right]}^{T}$ and **P**(0|0) = diag([1, 1, 1]), and ***ω***_*k*_ and ***ν***_*k*_ are mutually uncorrelated at each instant *k*.

The local estimate $\left({\hat{x}}_{i}\left(k|k\right),P_{i}\left(k|k\right)\right)$ is calculated by the cubature Kalman filter [38], and then propagated synchronously to the fusion center. We vary the correlation coefficient *ρ* ∈ [−0.7, 0.7] with step 0.1 and compare the ARMSEs of the state estimates over 500 time steps and 100 Monte Carlo runs. From the comparisons of the ARMSEs in Fig 5(B)–5(D), we can find that the proposed fusion algorithm GPA is consistently better than the other fusion methods, and the KLA, FCI, DCI, and WDCI have nearly the same performance while the GP behaves worst. Moreover, Table 3 reports the total (500 steps, 100 Monte Carlo runs, *ρ* = −0.1) computation time of all compared fusion algorithms, indicating the low computation cost of the GPA.

**Table 3:** Computation time (Seconds).

Fusion Method	KLA	FCI	GPA	GP	DCI	WDCI
Time	3.24	11.01	17.73	49.66	45.18	81.63

## 5 Conclusion

In this work, we propose a distributed estimation fusion method GPA under Gaussian assumptions. On the Gaussian submanifold with a fixed mean and a specified Lie algebraic structure, the GPA method fuses the geodesic projections of posterior PDFs in sense of minimizing the norm of the average displacement vector between a sought-for fused density and these projections. Simulation examples have illustrated that the GPA outperforms some existing fusion methods. It shows the significance of introducing the geodesic projection and Lie algebraic setting into distributed estimation fusion.
